# Studies on the Differentiation of Transient Chlorophyll *a* Fluorescence Signals in Papaya Plants Showing Symptoms and Without Symptoms in the Presence of PRSV-P and PMeV Viruses

**DOI:** 10.3390/plants14203208

**Published:** 2025-10-19

**Authors:** Weverton Pereira de Medeiros, Oeber de Freitas Quadros, Sabrina Garcia Broetto, José Aires Ventura, Diolina Moura Silva

**Affiliations:** 1 Nucleo de Estudos da Fotossintese, Universidade Federal do Espirito Santo, Vitoria 29075-010, ES, Brazil; wevertonmedeiros74@gmail.com (W.P.d.M.); sabroetto@yahoo.com.br (S.G.B.); 2 Nucleo de Biotecnologia, Universidade Federal do Espirito Santo, Vitoria 29040-090, ES, Brazil; oeberquadros@gmail.com (O.d.F.Q.); jose.a.ventura@ufes.br (J.A.V.); 3 Instituto Capixaba de Pesquisa, Assistencia Tecnica e Extensao Rural, Vitoria 29052-010, ES, Brazil

**Keywords:** papaya ringspot virus, sticky disease, chlorophyll *a* fluorescence, co-infection, non-destructive analysis

## Abstract

Viral infections represent a critical threat to cultivated plant species. In papaya cultivation, two viral diseases—papaya mosaic (caused by papaya ringspot virus type P—PRSV-P) and papaya sticky disease (caused by a virus complex of papaya meleira virus—PMeV, and papaya meleira virus—PMeV2)—are prevalent and capable of devastating entire plantations, incurring substantial economic losses. Current diagnostic practices rely on visual identification of symptoms and elimination of infected plants (roguing). Monitoring photosynthetic efficiency in orchards prone to PRSV-P and PMeV2 coinfection may allow early intervention, mitigating productivity losses and reducing fruit quality. This study aimed to evaluate chlorophyll *a* fluorescence as a biomarker for photosynthetic impairment and symptom severity in papaya infected with PRSV-P and/or PMeV2 and to explore the feasibility of early detection of the infection by these dual pathogens, as an exploratory study under field conditions. Chlorophyll *a* fluorescence revealed details about the physiology of plants coinfected with the complex of PMeV2 and PRSV-P: the electron motive force within PSII decreases in infected plants and in those without visual symptoms of infection, being proportional to the age and developmental stage of the plants. A slowdown in the multiple electron transfer turnover of PSII and a decrease in the efficiency of the redox reactions of photosystem I were observed in plants with or without visual detection of infection. The evidence generated suggests that the chlorophyll *a* fluorescence technique can be used to monitor the pathophysiological state of plants under biotic stress.

## 1. Introduction

The papaya (*Carica papaya* L.) is an economically important tropical fruit crop cultivated worldwide, with Brazil, India, Mexico, and the Dominican Republic among the leading producers. In Brazil, papaya production is concentrated in the state of Espírito Santo, where the crop plays a significant role in the agro-industrial economy. However, viral diseases pose a major threat to papaya cultivation, often leading to severe yield losses and reduced fruit quality [1].

Three types of viruses, which cause mosaic and sticky disease, are particularly destructive: papaya ringspot virus type P (PRSV-P), responsible for mosaic disease, and the papaya meleira virus complex (PMeV + PMeV2), which causes sticky disease. Both can infect plants at different developmental stages and spread rapidly under field conditions, sometimes remaining asymptomatic until flowering. Current control relies almost exclusively on visual detection and roguing—a method that is fast and low-cost but limited in sensitivity and often ineffective against asymptomatic infections. These limitations highlight the need for more sensitive, early, and non-destructive diagnostic approaches [2,3,4]. Visual symptoms of meleira, for example, typically become evident only after flowering, following a prolonged asymptomatic period, and early signs are often indistinguishable from other conditions, highlighting the need for alternative diagnostic approaches [5].

Papaya ringspot virus (PRSV-P) can infect plants at any stage of growth in a systematic manner. This virus, transmitted mainly by aphids [6], harbors an approximately 10.3 kb positive-sense, single-stranded RNA (ssRNA) genome containing a single open reading frame (ORF). This ORF encodes a large polyprotein that is processed into smaller proteins with diverse functions [7].

Papaya sticky disease is caused by a viral complex (PMeV) composed of a fusagravirus, also known as papaya meleira virus (PMeV), and the umbravirus-associated RNA (ulaRNA) virus papaya meleira virus 2 (PMeV2), which infects papaya plants in Brazil [8,9]. The PMeV viral complex usually infects the plant 6 to 8 months after seed germination, and the plant remains asymptomatic until flowering [5]. PMeV has a double-stranded RNA (dsRNA) genome of approximately 10 kb [10] or 12 kb. PMeV forms isometric, spherical particles approximately 38–42 nm in diameter, lacking external projections [11], containing two ORFs that encode a capsid protein (CP) and an RNA-dependent RNA polymerase (RdRp) [4]. PMeV2 is an ssRNA virus that lacks its own capsid and depends on the PMeV CP for encapsidation [4]. PMeV can even be detected in asymptomatic plants, and both viral RNAs can be detected in all symptomatic plants. In contrast, papaya ringspot virus type P (PRSV-P), which causes mosaic, is characterized by non-enveloped, flexuous, filamentous particles measuring about 760–800 nm in length and 12 nm in diameter, as reported by [12] and further confirmed by recent studies [13]. These structural differences reflect the viruses’ taxonomic divergence and have implications for their detection and transmission.

Plant viral infections progress through a sequence of events that begin with pathogen entry and replication, followed by systemic movement and interaction with host cellular processes. These interactions can alter plant physiology and metabolism before visible symptoms appear. Symptoms, such as chlorosis, mosaic, sticky latex, necrosis, or growth reduction, represent the external manifestation of the disease, but their presence depends on the virus, host genotype, and environmental conditions [14]. Understanding the temporal development of viral infection and symptom expression is essential for interpreting physiological indicators of stress, such as changes in chlorophyll *a* fluorescence.

There is extensive literature on viral infections in various plant species. Sugarcane yellow leaf virus (ScYLV) infection in sugarcane (*Saccharum* spp.) causes severe foliar symptoms and major changes in photosynthesis and metabolism [15]. Silencing the cytoplasmic receptor-like kinase gene TaRKL1 reduces photosynthetic capacity in wheat, showing its role in photosynthesis and H_2_O_2_ homeostasis [16]. Deg proteases are also essential for photoprotection and PSII repair in cereals [17]. In pepper (*Capsicum annuum*), the begomovirus chili leaf curl disease (ChiLCD) significantly impairs fruit and seed production [18]. Viral infestations, therefore, lead to major losses in many crops by disrupting metabolism and reducing plant vigor and reproduction.

Viral infections follow a series of steps—entry, replication, and systemic spread—that disturb host metabolism and physiology, often before symptoms appear. The disease refers to the physiological disorder, while symptoms like chlorosis, necrosis, or growth reduction depend on the virus, host genotype, and environment [14,19]. Early physiological changes, including photosynthetic alterations, offer key insights into plant–virus interactions and can be monitored non-invasively using chlorophyll *a* fluorescence [20,21].

In the papaya tree, previous studies have shown that during plant development under stress, changes in primary metabolism and photosynthesis occur [22,23,24,25]. In fact, PRSV-P causes severe chlorosis symptoms in the leaves of *C. papaya* plants. Ref. [26] used the rapid kinetics of Chl *a* fluorescence induction in healthy and PRSV-P-infected plants, and the results revealed damage to the electron transfer equilibrium on the acceptor side of PSII between Q_A_ and Q_B_ and to the size of the intersystem electron acceptor pool. Ref. [27] performed a virome network analysis in papaya orchards from two agroecological regions of Chiapas, Mexico; their findings suggested that management strategies need to be customized for each region and that visual assessment of papaya may be insufficient for PRSV, requiring diagnostic assays. They also warned that disease management strategies may not be successful if they are based solely on visual assessments and diagnostic assays for known individual viruses, as they found virus–virus interactions that could modify host symptoms.

After infecting plants, viral complexes alter physiological and biochemical processes, in addition to causing other tissue modifications, which can be visually detected in crops [28]. The infection compromises, for example, the functionality and morphology of chloroplasts, consequently altering the concentration of chlorophyll, which in turn affects plant growth and productivity [29,30].

Previous studies have shown that PRSV infections, whether single or mixed, are common in papaya and can produce multiple symptom patterns in the host [31].

Simultaneous viral infections have been previously reported [32,33,34,35]. The period of infection and the order in which viruses, in mixed infections, inoculate host tissues also significantly affect their interactions and can result in a range of responses [36].

Early detection of the disease will be highly valuable, considering that the average life expectancy of a papaya plant is 24 months and that from the tenth month, the plant begins to produce fruit continuously until the end of the cycle. Detection of the virus, either in the initial phase of vegetative growth or at the beginning of reproductive growth, would help producers avoid unnecessary investment in the crop.

Currently, producers use visual diagnosis to detect the infections caused by the viruses (PMeV and PRSV-P), as this is a fast and economical method. This method, however, has technical limitations. Papaya growers train field workers to visually identify plants with mosaic and/or sticky disease symptoms in papaya plantations and, upon detection, perform roguing. According to [9], without the implementation of this agricultural practice, these diseases would spread throughout the crop, resulting in total production losses. Although roguing has proven to be an effective strategy for controlling papaya viruses, its success depends on intensive monitoring. Even if “mosaic scouts” are adequately trained to identify disease symptoms early, other measures need to be implemented, such as vector control, crop rotation, and the adoption of phytosanitary technology protocols that ensure the absence of quarantine pests in papaya-importing countries. Thus, viral diseases in papaya are becoming increasingly complex, and for decades, papaya growers have faced the challenge of preventing and controlling infections caused by PRSV-P and the PMeV viral complex. Accurate and early molecular diagnosis [37,38] greatly facilitates the rapid and effective identification of these pathogens, allowing the development of more efficient management measures and control strategies aimed at mitigating the economic and agronomic impacts of these diseases on papaya crops. However, the method is invasive and has limited applicability. Proximal sensing methods, such as chlorophyll *a* fluorescence, make use of the possibility of evaluating physiological changes before changes in leaf structure occur, exhibiting greater sensitivity than that of other methods [39,40].

Monitoring the photosynthetic efficiency of *C. papaya* during different stages of development in orchards where both diseases can occur systematically will help in the early prevention of the disease, preventing farming losses caused by the severity of symptoms in infected green tissues and the associated reduction in crop productivity and in the economic value of the fruit. This study aimed to (a) use chlorophyll *a* fluorescence as a tool to evaluate the loss of photosynthetic efficiency and the severity of symptoms in green tissues infected by the mosaic virus and/or the papaya sticky disease virus and (b) explore the possibility of performing detection of the physiological alterations induced by both viral diseases in papaya plantations.

## 2. Results

**Figure 1:**
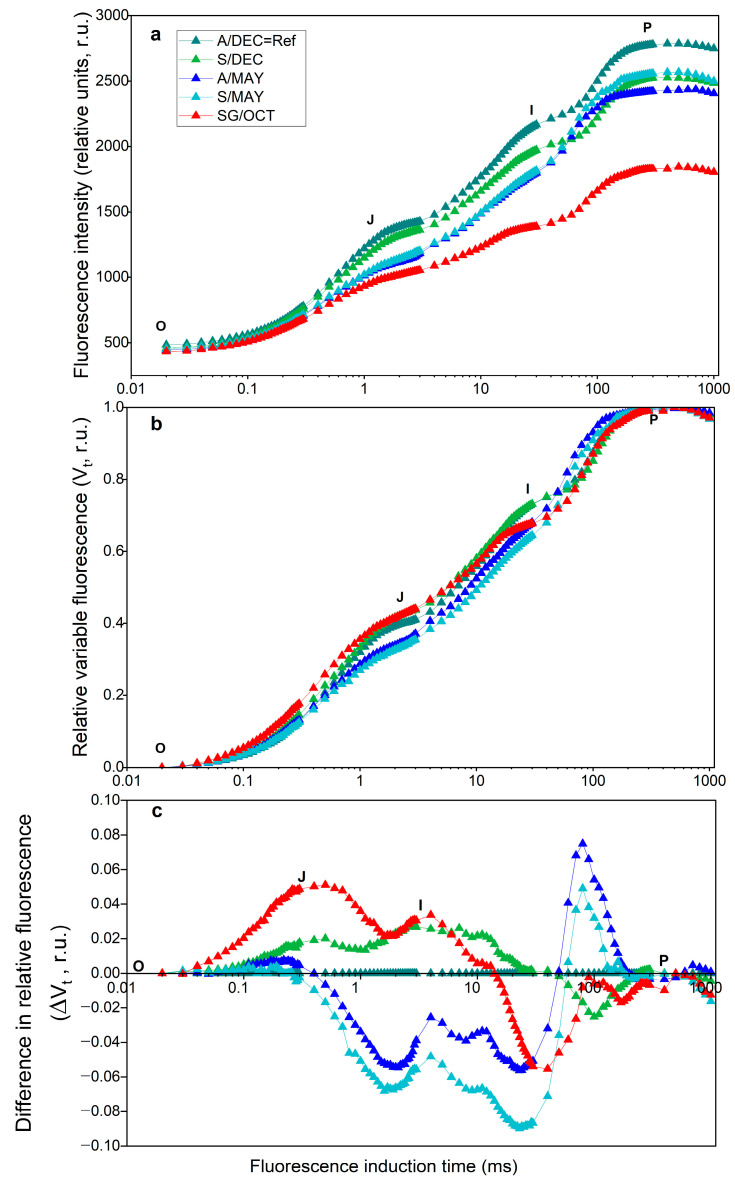
Polyphasic chlorophyll *a* fluorescence induction curves (OJIP). Fluorescence induction time is shown on a logarithmic scale. (**a**), relative variable fluorescence (Vt) after double normalization (**b**), and difference curves (ΔVt) (**c**) of papaya cv. Aliança leaves at different developmental stages and infection conditions. A/DEC: asymptomatic leaves, 8 months; S/DEC: symptomatic leaves, 8 months; A/MAY: asymptomatic leaves, 13 months; S/MAY: symptomatic leaves, 13 months; SG/OCT: severely symptomatic leaves, 24 months. Vt represents relative variable fluorescence normalized between minimum (F_0_) and maximum fluorescence (F_M_). ΔVt indicates the difference in Vt compared with the A/DEC reference. Time is shown on a logarithmic scale (ms). r.u., relative units. (*n* = 10).

The polyphasic chlorophyll *a* fluorescence transient (OJIP curve) revealed clear alterations in the photosynthetic performance of Carica papaya plants infected with papaya ringspot virus (PRSV-P) and/or papaya meleira virus complex (PMeV + PMeV2) at different developmental stages (Figure 1a). After double normalization of the fluorescence data (Figure 1b), differences in the relative variable fluorescence (ΔVt) became evident between asymptomatic and symptomatic plants (Figure 1c).

Negative differences appeared in the J–I phase in symptomatic plants at 13 months (S/MAY), while positive differences occurred in the O–J and J–I phases in symptomatic plants at 8 months (S/DEC) and in severely symptomatic plants at 24 months (SG/OCT). Although a slight decrease in maximum fluorescence (P-step, ~300 ms) was observed in infected plants, the normalization of the fluorescence transients allows a clearer comparison of kinetic differences among treatments.

**Figure 2:**
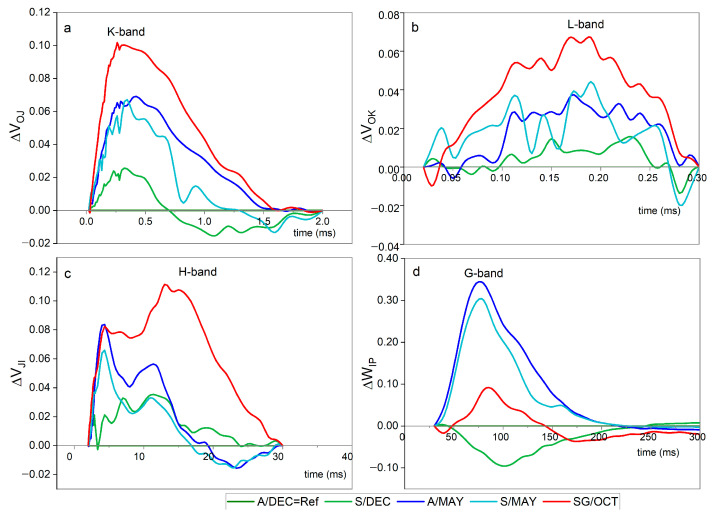
Differential curves (ΔVt) of chlorophyll *a* fluorescence transients in papaya cv. Aliança leaves under different infection conditions: A/DEC = Ref (asymptomatic, 8 months), A/MAY (asymptomatic, ~13 months), S/DEC (symptomatic, 8 months), S/MAY (symptomatic, ~13 months), and SG/OCT (severely symptomatic, 24 months). Asymptomatic plants (A/DEC) were used as the reference (ΔVt = 0). (**a**) K-band (~300 µs) indicates oxygen-evolving complex (OEC) destabilization; (**b**) L-band (~150 µs) reflects impaired connectivity among PSII units; (**c**) H-band (~20 ms) shows slowed electron transfer from PSII to the plastoquinone pool; and (**d**) G-band (~100 ms) indicates reduced efficiency of electron transfer to PSI acceptors. Positive bands reveal progressive photosynthetic impairment associated with viral infection, even before visible symptoms. Time is plotted on a logarithmic scale (µs–ms), and ΔVt is expressed in relative units (r.u.) (*n* = 10).

In severely symptomatic plants (SG/OCT), pronounced positive L- and K-bands were detected (Figure 2), indicating impaired connectivity among PSII units and a disturbance in the oxygen-evolving complex (OEC).

These changes indicate stress response but require complementary analyses for mechanistic confirmation.

Similar L- and K-band signatures were also observed, though with lower amplitude, in asymptomatic and symptomatic plants sampled at 13 months. The presence of these bands suggests structural and functional stress in PSII even in the absence of visible disease symptoms.

**Figure 3:**
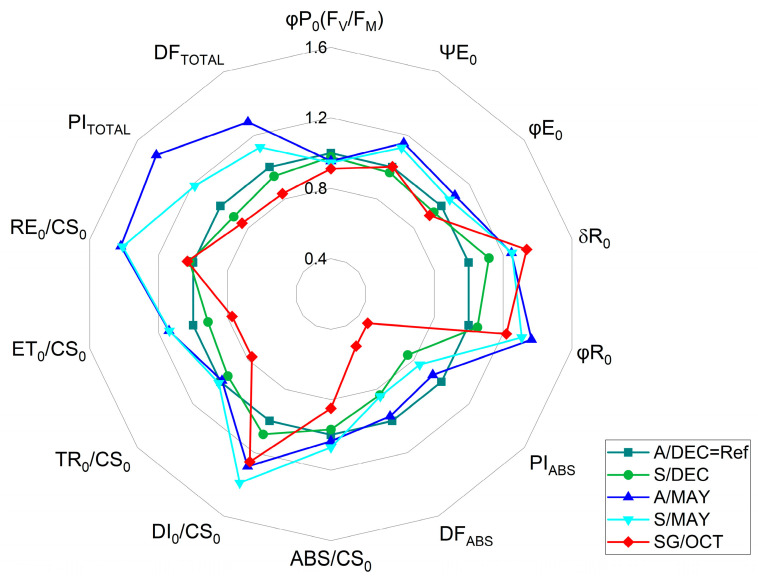
Radar plot of selected JIP-test parameters derived from chlorophyll *a* fluorescence measurements of papaya cv. Aliança leaves under different infection conditions: A/DEC (asymptomatic, 8 months), A/MAY (asymptomatic, ~13 months), S/DEC (symptomatic, 8 months), S/MAY (symptomatic, ~13 months), and SG/OCT (severely symptomatic, 24 months). Values are normalized to the asymptomatic reference (A/DEC = Ref = 1.0). (*n* = 10). Abbreviations: φP_0_ (=F_v_/F_M_), maximum quantum efficiency of PSII in dark-adapted leaves; ΨE_0_, probability that an absorbed photon moves an electron beyond Q_A_; φE_0_, quantum yield of electron transport; δR_0_, relative electron flux per reaction center; φR_0_, efficiency of reduction in the final PSI acceptor; PI_ABS_, performance index on absorption basis; DF_ABS_, (density of absorbed photons per unit leaf area); PI_TOTAL_, total performance index; ABS/CS_0_, absorbed energy flux per cross section; DI_0_/CS_0_, dissipated energy flux per cross section; TR_0_/CS_0_, trapped energy flux per cross section; ET_0_/CS_0_, electron transport flux per cross section; RE_0_/CS_0_, electron flux reducing the end acceptors at the PSI acceptor side per cross section; DF_TOTAL_, total photon flux density incident on the leaf surface (total number of photons reaching the leaf per unit area. Statistical analyses of the raw data are provided in Supplementary Table S2.

Analysis of JIP-test parameters showed significant differences among treatments (Figure 3). Younger plants (A/DEC) exhibited the highest total performance index (PI_TOTAL_), total driving force (DFTOTAL), and energy flux to the PSI acceptor side (RE_0_/CS_0_). In contrast, PI_TOTAL_ declined progressively from asymptomatic (A/MAY) to symptomatic (S/MAY and S/DEC) plants, reaching the lowest values in severely symptomatic plants (SG/OCT). The maximum PSII performance index (PI_ABS_) decreased gradually with disease progression, while energy dissipation per reaction center (DI_0_/RC) increased with plant age and symptom severity.

The analysis of the maximum quantum yield of PSII photochemistry (φP_0_ ≡ F_V_/F_M_) revealed a progressive decline relative to asymptomatic reference plants (A/DEC = 1.0). In symptomatic plants sampled at 8 months (S/DEC), φP_0_ decreased by 2.18%, while asymptomatic plants at 13 months (A/MAY) showed a 4.54% reduction. Symptomatic plants at the same stage (S/MAY) exhibited a 5.47% decrease, and the lowest value was observed in severely symptomatic plants at 24 months (SG/OCT), with an 8.91% reduction compared to the control (For further details, see Supplementary Table S2). These results demonstrate a gradual loss of maximum PSII potential associated with disease progression and symptom severity.

No significant differences in total chlorophyll content (SPAD index) were detected between asymptomatic and symptomatic plants at 8 or 13 months. However, plants showing severe symptoms at 24 months exhibited significantly reduced chlorophyll levels (Table 1), consistent with advanced physiological decline.

**Figure 4:**
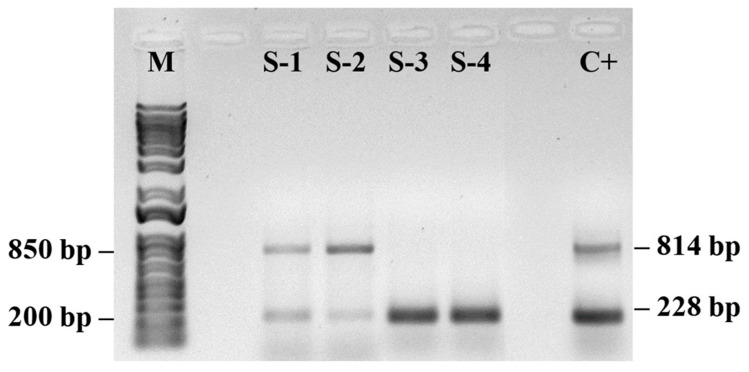
PCR amplification products for detection of papaya meleira virus (PMeV2; 814 pb) and papaya ringspot virus (PRSV-P; 228 pb) in papaya samples. (M) 1 Kb Plus DNA Ladder (Thermo Fisher Scientific, Waltham, MA, USA) and a control sample (C+) containing both PMeV2 and PRSV-P viruses. (*n* = 10).

The S/DEC samples presented varying patterns of PMeV2 and PRSV-P gene presence (Figure 4). The analyzed samples presented either both viruses (samples S-1 and S-2) or only PRSV-P (samples S3 and S4).

Agarose gel electrophoresis (1%) showed two distinct profile patterns for the plants. Samples S1 and S2 presented two well-defined bands consistent with the sizes of the PMeV2 (814 bp) and PRSV-P (228 bp) amplicons, indicating coinfection, whereas the symptomatic plant samples S3 and S4 showed a single, well-defined band at the position corresponding to ~200 bp, compatible with the expected size for the PRSV-P (amplified with specific primers). There were no additional bands, indicating the absence of coinfection. The positive control confirmed the specificity of the reaction.

In plants showing co-infection, the physiological alterations were more pronounced, including higher positive L- and K-band amplitudes, lower PI*_TOTAL_*, and reduced RE_0_/CS_0_ values. These plants also exhibited increased DI_0_/RC compared with plants infected by a single virus, indicating a stronger disruption of PSII photochemistry and electron transport.

**Figure 5:**
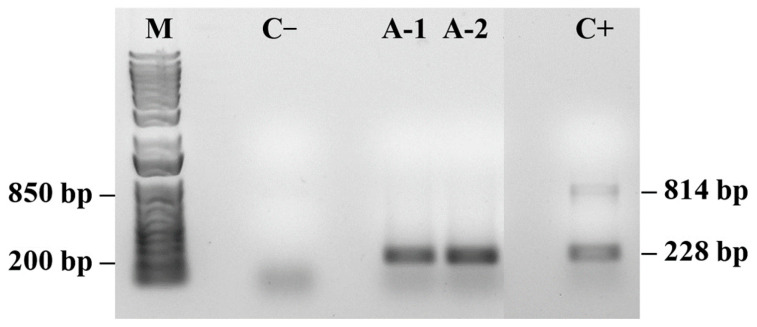
PCR detection of papaya meleira virus (PMeV2; 814 pb) and papaya ringspot virus (PRSV-P; 228 pb) in asymptomatic papaya plants. (M) 1 Kb Plus DNA Ladder (Thermo Fisher Scientific). Negative control (C−) shows no amplification. Positive control (C+) contains both viral targets. (*n* = 10).

PRSV-P was detected even in asymptomatic plants sampled at 13 months (A/MAY) (Figure 5). Although these plants showed no visible disease symptoms, their fluorescence parameters differed from those of younger asymptomatic plants (A/DEC), including slightly elevated RE_0_/CS_0_ and δR_0_ values and the presence of a weak positive L-band. These results indicate that viral infection was already affecting photosynthetic performance before symptom expression.

**Figure 6:**
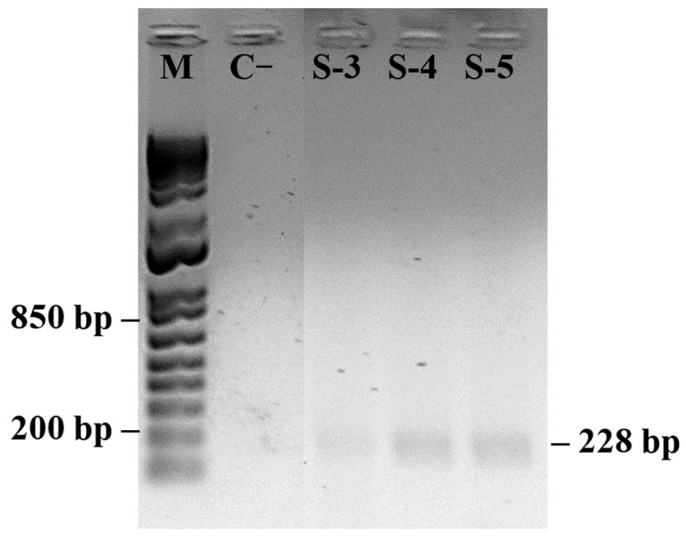
PCR detection of papaya meleira virus (PMeV2; 814 pb) and papaya ringspot virus (PRSV-P; 228 pb) in symptomatic papaya plants. (M) 1 Kb Plus DNA Ladder (Thermo Fisher Scientific). Negative control (C−) shows no amplification. (*n* = 10).

**Figure 7:**
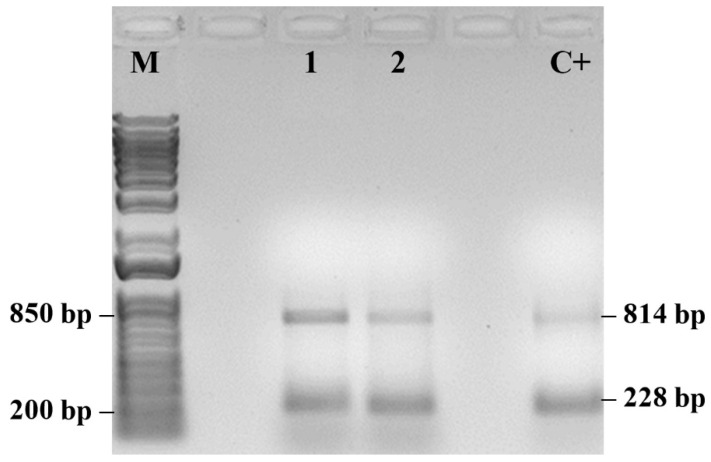
PCR detection of papaya meleira virus 2 (PMeV2; 814 pb) and papaya ringspot virus (PRSV-P; 228 pb) in symptomatic papaya plants. (M) 1 Kb Plus DNA Ladder (Thermo Fisher Scientific). Positive control (C+). (*n* = 10).

Symptomatic plants sampled in May (S/MAY) showed the presence of PMeV2 as the predominant viral component (Figure 6). These plants presented higher PI_TOTAL_ values than severely symptomatic plants (SG/OCT) but lower than those of asymptomatic plants (A/DEC), indicating intermediate physiological performance during infection progression. Molecular analysis of samples used in the fluorescence measurements confirmed the presence of both viral pathogens in symptomatic plants (Figure 7).

Co-infected plants showed the most severe deviations in fluorescence parameters, including lower PIABS, δR_0_, and φR_0_, and higher DI_0_/RC, compared with plants infected with a single virus. These results demonstrate that mixed infections are associated with more severe impairment of the photosynthetic apparatus.

The detection of PRSV-P and PMeV by PCR confirms the presence of viral pathogens in the plants analyzed for chlorophyll *a* fluorescence, supporting the physiological changes observed in OJIP parameters.

## 3. Discussion

In our experiment, we obtained specific ΔVt values that provided valuable information about the physiological state of uninfected plants (based on visual diagnosis) and those that already presented symptoms of the infection caused by the papaya ringspot virus (PRSV-P) and the papaya meleira virus (PMeV), allowing a comparison with plants that presented severe symptoms of infection (SG/OCT).

Changes in the photosynthetic components that trigger and fine-tune the responses of infected plants to biotic stress have been reported by several authors. Viral infections can directly affect chloroplast function through interactions between viral proteins and components of the photosynthetic machinery, leading to the fluorescence alterations observed in this study. For example, silencing of the 33 kDa subunit of the oxygen-evolving complex (OEC) increases virus replication and compromises PSII activity [41], while TMV flavum reduces OEC protein levels and disrupts electron transport [42]. Interactions between viral proteins and PsbO also induce chloroplast structural changes [43], which may explain the increased K-band associated with OEC destabilization. Moreover, virus-induced reorganization of thylakoid membranes and pigment–protein complexes reduces excitation energy transfer efficiency [44], contributing to changes in the OJIP transient. Although abiotic stressors can produce similar patterns, future studies including abiotic controls are needed to validate the specificity of fluorescence-based viral detection.

In 2017, Ref. [45] reported a relatively high content of photosynthesis-related proteins in PMeV-infected plants, confirming the involvement of the viral complex (PMeV) in photosynthesis. Ref. [25] quantified cellular proteins during the development of infected papaya plants and reported an increase in the efficiency of energy flow in the photosystems. We detected differences in the kinetics of chlorophyll a fluorescence emission and observed that these differences occurred at the same points in the OJIP curve; however, depending on the development stage of the infected plants, the intensities were different.

The observed increase in the K-band, particularly in highly infected plants (SG/OCT) and 13-month-old plants (A/S MAY), indicates early destabilization of the OEC. This pattern suggests that viral interference in photosystem II occurs before visible symptom manifestation, reflecting early stages of viral replication [46]. This statement was confirmed by the results we obtained when calculating the JIP test parameters [47,48]. There was little change in the energy flux in PSII (PI_ABS_). Only the SG/OCT plants showed a sharp decrease. The captured energy flux (TR_0_/CS_0_) and the intersystem electron transport flux (ET_0_/CS_0_) did not differ between symptomatic and asymptomatic plants, except in severely symptomatic plants, where a sharp decrease was observed. The energy dissipation flux (DI_0_/CS_0_) gradually increased from younger to older plants. These results confirmed the presence of a positive K-band, which was higher in leaves with severe symptoms in October (SG/OCT) [49,50].

Consistently, the progressive decline in the maximum quantum yield of PSII photochemistry (φP_0_) across sampling points further supports this interpretation. This pattern reflects the progressive impairment of PSII reaction centers and the oxygen-evolving complex described under biotic stress conditions [51,52,53], and reinforces the utility of φP_0_ (Fv/F_M_) as a sensitive indicator of functional changes in the photosynthetic apparatus during virus–plant interactions.

At the molecular level, viral proteins may directly interact with PSII components, including PsbO and D1, leading to structural destabilization and altered electron transport. Such interactions provide a mechanistic explanation for the early fluorescence changes observed even in asymptomatic plants, as previously reported for TMV [41,42] and Alternanthera mosaic virus [43], and are consistent with proteomic evidence of photosynthesis-related protein alterations in PMeV-infected papaya [25,45].

The presence of L-bands in asymptomatic (A), symptomatic (S) and severely symptomatic (SG) plants was consistently positive compared with that in the reference plants (A/DC). In all the treatments, the increase in each curve followed the same pattern as the K-band, with smaller curves for asymptomatic leaves of younger plants and larger curves for fully symptomatic leaves in October. Our results confirm the results of [25], who reported an accumulation of photosynthetic proteins followed by an accumulation of proteins involved in the synthesis of wall precursors. The authors suggest that *C. papaya* plants “struggle” to maintain the integrity of the laticifer walls and fail to do so after 4 months, leading to latex exudation.

The final stage of the electron transport chain, represented by the chlorophyll *a* fluorescence transient that reflects the efficiency of electron transfer from plastoquinol (PQH_2_) to PSI acceptors (δR_0_), was significantly influenced by both viral presence and plant age. Similar effects were reported in young maize plants under salt stress [49]. The higher values observed for the energy dissipation flux per cross section (DI_0_/CS_0_), the efficiency of electron transport from reduced plastocyanin to the PSI acceptor side (δR_0_), the quantum yield of electron transport from Q_A_ to the terminal PSI acceptors (φR_0_), the reduction flux through the cross section at t = 0 (RE_0_/CS_0_), and the total driving force based on the excited cross section (DF_TOTAL_) suggest that these parameters are more sensitive indicators of subtle differences than the multivariate performance indices (PI_ABS_ and PI_TOTAL_).

The H and G bands reflect later stages of photosynthetic impairment, with slowed electron transfer between PSII and PSI. Their presence in symptomatic plants indicates that viral effects progress gradually, allowing the association of fluorescence dynamics with intermediate and late stages of viral replication [54].

Refs. [50,51] described the H- and G-bands as two distinct peaks between the I and P steps, corresponding to multiple turnover deceleration events within the electron transport chain: the second reduction of Q_B_^−^ to Q_B_^2−^ (H-band at 20 ms) and the formation of a second protonated quinone acceptor, PQH_2_ (G-band at 100 ms). In our results, the appearance of a positive H-band is particularly evident and likely reflects multiple turnovers slowing electron transfer from PSII to the PQ pool. This interpretation is supported by the concurrent decreases in PI_ABS_ and DF_ABS_ observed in May and SG plants. The formation of a positive G band indicates reduced efficiency of electron transfer to the acceptor side of PSI, suggesting impaired redox reactions possibly caused by viral infection (papaya meleira virus or papaya ringspot virus type P). Notably, a negative G-band was observed only in leaves of 8-month-old plants.

It should be noted that measurements of OJIP-curves provide indirect information on photosystem II performance and plant physiological status. While valuable for rapid screening of stress responses, OJIP-based parameters alone are insufficient for unambiguous conclusions regarding the molecular mechanisms of viral infection. Therefore, results should ideally be complemented with biochemical or biophysical measurements, such as D1 and PsbO protein content or O_2_-evolving activity, as reported in previous studies [52,53]. Despite this limitation, the OJIP-test remains a sensitive, non-invasive tool for early detection of physiological alterations induced by viral infection.

Complementary findings from the JIP test revealed that viral infection disrupts chlorophyll biosynthesis, leading to leaf yellowing—consistent with the observations of [15] in ScYLV-infected sugarcane plants. Similarly, PRSV-P induces severe mosaic symptoms in papaya and causes structural alterations in PSII, including increased chlorophyll a fluorescence polarization, suggesting pathogen-induced thylakoid membrane transformation. Such modifications in the physical state of the thylakoid affect pigment–protein topology and impair energy transfer from carotenoids to chlorophylls [26]. Our results corroborate these findings, indicating that both PRSV-P and PMeV alter chloroplast structure and photochemical function, resulting in reduced photon utilization and PSII activity. These effects are reflected in changes in the O–J phases (appearance of K and L bands), the IP phase (H and G bands), and the electron transfer between Q_A_ and Q_B_, evidenced by an increase in the PSI electron acceptor pool (δR_0_, RE_0_/CS_0_). Consequently, higher PI_TOTAL_ values were observed in both younger asymptomatic and older symptomatic plants.

Even in asymptomatic plants (A/MAY), alterations in K and L-bands indicate that viral replication already affects OEC function and photosynthetic efficiency, demonstrating that fluorescence can detect infection before visible symptoms. High PSI efficiency (δR_0_, φR_0_, RE_0_/CS_0_, DF_TOTAL_) detected by the chlorophyll *a* fluorescence method may already indicate alterations before symptoms are noticeable, as observed in A/MAY plants in which, although the presence of the PMeV virus was confirmed, visible symptoms were not observed.

Another relevant factor for the presence of the virus even in asymptomatic plants is the low initial viral load, which may limit the immediate impact on chloroplast organization and chlorophyll production, as may be the case in young asymptomatic plants evaluated 13 months after planting (A/MAY). In addition, some viruses have the ability to modulate the response to oxidative stress, reducing the damage caused in the early stages of infection [54]. Even with this regulation, the efficiency of PSII may be compromised, resulting in changes in chlorophyll *a* fluorescence, even if visible symptoms only appear later.

The increased oxidative stress in plants with mixed infections may result not only from general metabolic disruption but also from direct viral protein interactions with chloroplast enzymes, impairing photoprotective mechanisms and promoting energy dissipation as heat, as previously highlighted for virus-infected papaya [44,55,56].

PCR detection of PRSV-P and PMeV (Figure 4, Figure 5, Figure 6 and Figure 7) confirmed the presence of viral pathogens in the plants analyzed for chlorophyll *a* fluorescence, supporting the physiological alterations observed in the OJIP parameters. The coincidence between PCR results and specific fluorescence band changes indicates that these patterns reflect distinct stages of the viral cycle and their effects on photosynthesis. Coinfection by multiple viruses may intensify oxidative stress, increasing energy dissipation as heat, which is detectable through fluorescence measurements. These results reinforce that the observed changes in PSII and PSI performance are attributable to viral infection rather than other environmental or biotic factors. Although this study was not intended to establish a diagnostic protocol, it demonstrates the potential of chlorophyll *a* fluorescence as an early physiological indicator of infection. Further studies integrating fluorescence analyses with broader molecular and biochemical assays will help validate and refine this approach for early detection and for elucidating how mixed viral infections influence plant metabolism [44,55,56].

It is important to emphasize that the physiological alterations detected by chlorophyll *a* fluorescence were corroborated by PCR-based detection of PRSV-P and PMeV in the same samples. The PCR results confirm that changes in fluorescence parameters are associated with viral infection. Therefore, our findings should be interpreted as an exploratory demonstration of early physiological responses rather than a validated diagnostic protocol. Further validation against established detection methods such as ELISA and multiplex RT-PCR will be essential before this approach can be reliably used for early virus detection in the field.

The results further indicate that the PRSV-P and PMeV infections can be used to understand the complexities involved at the molecular level, and the experiments, particularly the measurement of induction kinetics, can be used for rapid assessment of the pathophysiological state of plants subjected to biotic stress.

## 4. Materials and Methods

### 4.1. Experimental Area and Plant Material

This study was carried out on the papaya cv. Aliança, and the analyses were performed at two sites over three distinct periods of plant development. The first data collection was carried out in October 2022 in Rio Quartel, Linhares, ES (19°31′21.8″ S 40°13′01.1″ W/−19.522716, −40.216967). The plants were in the fruiting stage at approximately 24 months after planting and presented severe symptoms of papaya mosaic and sticky diseases. Immediately after data and sample collection, the plants were exterminated following Normative Instruction No. 17 of the Ministry of Agriculture, Livestock and Food Supply (MAPA), on 27 May 2010. The analyses were performed on 10 plants per treatment.

The 2nd and 3rd collections were carried out in the municipality of Guaraná, Aracruz, ES (19°38′22.5” S 40°15′04.6” W/−19.639578, −40.251265). In December 2022 and May 2023, samples were collected from healthy plants (A) and from symptomatic plants (S). During the second collection, the plants were 8 months old and in their first fruiting. During the third collection, the crop was sampled at 13 months after planting. The treatment groups described in the text were as follows: A/DEC = asymptomatic leaves from plants 8 months after planting (sampled in December 2022), A/MAY = asymptomatic leaves from plants 13 months after planting (sampled in May 2023), S/DEC = symptomatic leaves from plants 8 months after planting (sampled in December 2022), S/MAY = symptomatic leaves from plants 13 months after planting (sampled in May 2023), and SG/OCT = leaves with severe viral infection symptoms from plants 24 months after planting (sampled in October 2022). Leaf samples from healthy plants were also collected from plantations where care was taken to detect the first symptoms of viral infection daily.

### 4.2. Transient Chlorophyll a Fluorescence

To analyze the transient fluorescence of chlorophyll *a*, a portable fluorometer (HandyPEA, Hansatech Instruments, King’s Lynn, Norfolk, UK) was used. Analyses were performed on fully expanded leaves in the early morning hours to avoid the inhibitory effects of high temperature and light on photosynthetic reactions. Measurements were performed on 10 plants per experiment, with five readings taken per leaf. Leaves were acclimated in the dark for 40 min (at which time all photosystem reaction centers were fully oxidized). After dark acclimation, the leaves were exposed to a saturating amount of red light (3000 μmol m^−2^ s^−1^). The fluorescence intensities were recorded between 20 μs and 1 s, where 20 μs was the timepoint for the initial fluorescence (F_0_) and ±300 ms was the maximum fluorescence (F_M_). The fluorescence intensity data were normalized to the relative fluorescence (Vt), where Vt = (Ft − F_M_)/(F_M_ − F_0_). The differences among plants from the three data collections were calculated using the results for A/DEC plants (asymptomatic leaves of plants 8 months after planting, sampled in December 2022) as a reference to obtain ΔVt = F (treatment) − F (reference) [57]. The established parameters were calculated on the basis of the fluorescence intensities via the JIP test [47] (Supplementary Table S1).

### 4.3. Chlorophyll Index

The total chlorophyll index was determined with the same leaves used for photochemical performance measurements using a portable chlorophyll meter (SPAD-502 Plus, Konica Minolta Optics Inc., Osaka, Japan). This instrument provides a convenient and low-cost method for measuring the relative chlorophyll content of a leaf sample using dual-wavelength (at 620 and 940 nm) optical absorbance measurements [58].

### 4.4. Molecular Diagnostics

The same leaf samples from ten plants, which were used to determine chlorophyll indices and chlorophyll *a* fluorescence, were also used to assess the presence of viruses associated with mosaic and bacterial blight symptoms, following established molecular methodologies described in previous studies [8,38,59]. For molecular analysis, leaf tissue from each plant was collected and pooled to ensure sufficient material and reproducibility of RNA extraction. Total RNA was extracted using TRIzol™ Reagent (Invitrogen, Carlsbad, CA, USA), following the manufacturer’s instructions. PCR amplification was performed using Taq DNA Polymerase (Invitrogen, Carlsbad, CA, USA), following the manufacturer’s instructions. Additionally, leaf samples from asymptomatic plants at the cultivation sites, which were subjected to daily surveillance, were collected for early detection of viral infection symptoms.

### 4.5. Experimental Design and Statistical Analysis

The experiment was performed with a completely randomized design with two treatment groups, asymptomatic plants and symptomatic plants, evaluated at three stages of development: 8 months after planting (December 2022), 13 months after planting (May 2023) and 24 months after planting (October 2022). Each plant was designated as an independent experimental unit.

The collected data were subjected to analysis of variance (ANOVA) to assess treatment effects. When ANOVA yielded significant F values (α = 0.05), mean comparisons were conducted via Tukey’s honestly significant difference (HSD) test at the 5% probability level, using INFOSTAT statistical software (2020, Grupo Infostat, Córdoba, Argentina).

## 5. Conclusions

Chlorophyll *a* fluorescence measurements revealed detailed physiological responses of papaya plants under PMeV and PRSV-P coinfection. Changes in PSII electron transport (DFABS) and in multiple turnover steps (J–I, I–P; positive K, L, H, and G bands), and decreases in PSI efficiency (δR_0_, RE_0_/CS_0_), were detected not only in symptomatic plants but also in asymptomatic individuals, with patterns influenced by plant age and developmental stage. These findings indicate that subtle alterations in the performance of photosystems II and I may precede visible symptoms of infection.

Although infected young plants initially exhibit an increase in PSI efficiency and overall performance (PI_TOTAL_), PSII is affected early, resulting in reduced PI_ABS_ as infection progresses. Therefore, chlorophyll *a* fluorescence provides a sensitive, rapid approach to monitor the pathophysiological state of plants under biotic stress, potentially allowing early detection of viral infection, providing an exploratory physiological indicator. Nevertheless, we emphasize that OJIP-based screening should be complemented with molecular or biochemical assays to confirm infection and fully understand the underlying mechanisms.

## Figures and Tables

**Table 1:** Total chlorophyll content (SPAD index) of papaya cv. Aliança with severely symptomatic (SG), asymptomatic (A) and symptomatic (S) leaves and fruits, measured in October 2022, December 2022 and May 2023 on rural properties in the rural area of Aracruz and Linhares, ES. Mean ± SE, (*n* = 10).

	SG/OCT	A/DEC	S/DEC	A/MAY	S/MAY
Chla	39.8 ± 4.76	52.55 ± 5.10	48.2 ± 4.52	49.55 ± 2.50	48.2 ± 1.61

## Data Availability

Data are contained within the article and Supplementary Materials.

## Supplementary Materials

The following supporting information can be downloaded at https://www.mdpi.com/article/10.3390/plants14203208/s1.
