# Supplementary material for: Studies on the Differentiation of Transient Chlorophyll a Fluorescence Signals in Papaya Plants Showing Symptoms and Without Symptoms in the Presence of PRSV-P and PMeV Viruses
Source: Plants (Basel). 2025 Oct 19;14(20):3208. doi: 10.3390/plants14203208 (PMC12567204; doi:10.3390/plants14203208)
Supplement: Supplementary file 1 [file plants-14-03208-s001.zip › Supplementary material Table S1.pdf]

Studies on the differentiation of transient chlorophyll *a* fluorescence signals in papaya plants showing symptoms and without symptoms in the presence of PRSV-P and PMeV viruses

Table S1. Glossary, definition of terms and formulas used in the JIP test for the analysis of chlorophyll *a* fluorescence emitted by dark-adapted photosynthetic samples (modified by [1] and [2]).

Parameters	***Synonyms, Formula***	***Definition***
F_0	$F_{20\mu s}$	Initial fluorescence; minimal reliable recorded F (at O-step), taken commonly as the F emitted when all RCs are open
F_J	J step; F_{2ms}	J-step fluorescence intensity (at 2 ms)
F_I	I step; F_{30ms}	Fluorescence intensity at step I (30 ms)
F_M	F_P, P-step	Maximum fluorescence in stage P, when all CRs are closed.
F_V	F_M-F_0	Maximal variable fluorescence
φP_0	$= TR_0 /ABS$	maximum quantum yield for primary photochemistry
ψE_0	ET_0/TR_0	Quantum yield of electron transport
φE_0	$= ET_0/ABS$	Quantum yield of electron
δR_0	$= RE_0 - ET_0$	Efficiency /probability with which an electron from the intersystem electron carriers is transferred to reduce end electron acceptors at the PSI acceptor side (RE)
φR_0	$= RE_0/ABS$	Quantum yield for reduction of end electron acceptors at the PSI acceptor side (RE)
DI_0/RC	$ABS/RC - TR_0/RC$	Dissipation flux per active RC
ABS/CS_0	$JABS/CS_0\ ABS/CS \approx F_0$	Absorbed energy flux per excited cross-section (=CS) at a time t = 0
DI_0/CS_0	$(ABS/CS_0) - (TR_0/CS_0)$	Energy dissipation flow by CS
TR_0/CS_0	$\Phi_0 \cdot (ABS/CS_0)$	Captured energy flow per excited cross-section
ET_0/CS_0	$\Phi_0 \cdot \Psi_0 \cdot (ABS/CS_0)$	Flux of electrons from Q_A^- to PQ per cross section
RE/CS_0	RE_0 /CS_0	Reduction flux of final acceptors on the electron acceptor side of the FSI by CS at t = 0
Vt	$= (F_t - F_M)/(F_M - F_0)$	relative variable fluorescence at time t (normalization on $F_M - F_0$)

L-step	F_{100}, F_{150}, L_{band}	Fluorescence observed at 100 or 150 µs
K-step	F_{300}, V_K, V_{OJ300}, K_{band}	Fluorescence observed at 300 µs
H-step	H_{band}	Fluorescence observed at 20 ms, and represents the second reduction of Q_B^- to Q_B^{2-}
G-step	G_{band}	formation of a second protonated quinone acceptor, PQH_2 at 100 ms
PIabs	$(RC/ABS)\cdot[\varphi P_O/(1\varphi P_O)]\cdot[\Psi_{Eo}/(1-\Psi_{Eo})]$	Performance index on absorption basis
PI_{TOTAL}	$PIABS\cdot[\delta Ro/(1-\delta Ro)]$	Performance index for energy conservation from photons absorbed by *PSII* antenna, until the reduction of *PSI* acceptors
DF_{abs}	$Log(PI_{ABS})$	Driving force on absorption basis
DF_{TOTAL}	$Log(PI_{total})$	driving force (potential) for energy conservation from photons absorbed by PSII until the reduction of PSI end electron acceptors

References:

1. Strasser, R.J.; Tsimilli-Michael, M.; Srivastava, A. Analysis of the fluorescence transient In**:** Papageorgiou, G. C.; Govindjee (eds.), Chlorophyll fluorescence: A signature of photosynthesis. Advances in Photosynthesis and Respiration Series. Springer: Dordrecht, p. 321-362, 2004.
2. Tsimilli-Michael M.; Strasser, R.J. (2008) *In Vivo* Assessment of Stress Impact on Plants' Vitality: Applications in Detecting and Evaluating the Beneficial Role of Mycorrhization on Host Plants. In: Varma A (ed) Mycorrhiza: State of the Art, Genetics and Molecular Biology, Eco-Function, Biotechnology, Eco-Physiology, Structure and Systematics, 3rd edition (ISBN: 978-3-540-78824-9), Springer, pp 679-703
