# Supplementary material for: Studies on the Differentiation of Transient Chlorophyll a Fluorescence Signals in Papaya Plants Showing Symptoms and Without Symptoms in the Presence of PRSV-P and PMeV Viruses
Source: Plants (Basel). 2025 Oct 19;14(20):3208. doi: 10.3390/plants14203208 (PMC12567204; doi:10.3390/plants14203208)
Supplement: Supplementary file 1 [file plants-14-03208-s001.zip › Supplementary material Table S2.pdf]

Studies on the differentiation of transient chlorophyll *a* fluorescence signals in papaya plants showing symptoms and without symptoms in the presence of PRSV-P and PMeV viruses

Table S2. Chlorophyll *a* fluorescence parameters in leaves of papaya plants collected from healthy plants (A) and from symptomatic plants (S). A/DEC=Ref (asymptomatic, 8 months), A/MAY (asymptomatic, ~13 months), S/DEC (symptomatic, 8 months), S/MAY (symptomatic, ~13 months), and SG/OCT (severely symptomatic, 24 months).

Treatment/parameter	φP_0	ψE_0	φE_0	$\delta(R_0)$	φR_0	PI_{ABS}	DF_{ABS}	ABS/CS_0	DI_0/CS_0	TR_0/CS_0	ET0/CS0	RE_0/CS_0	PI_{TOTAL}	DF_{TOTAL}
**A/DEC=Ref**	0.8396 ±0.012 (a)	0.6038 ± 0.038 (a)	0.5071 ± 0.032 (a)	0.4319 ± 0.043 (b)	0.2152 ± 0.027 (b)	5.773 ± 1.921 (a)	0.713 ± 0.143 (a)	454.90 ± 36.94 (a)	73.36 ± 10.34 (c)	381.54 ± 25.68 (a)	230.18 ± 17.52 (b)	99.22 ± 10.93 (b)	4.415 ± 1.520 (c)	0.631 ± 0.166 (a)
**S/DEC**	0.8242 ± 0.019 (b)	0.5795 ± 0.050 (b)	0.4778 ± 0.039 (b)	0.4567 ± 0.046 (b)	0.2090 ± 0.024 (b)	4.366 ± 1.289 (b)	0.603 ± 0.117 (b)	441.95 ± 44.81 (a)	79.47 ± 13.89 (c)	362.48 ± 31.89 (b)	211.66 ± 21.87 (c)	95.42 ± 12.13 (b)	3.607 ± 1.183 (c)	0.532 ± 0.135 (b)
**A/MAY**	0.7993 ± 0.040 (c)	0.6776 ± 0.060 (a)	0.5417 ± 0.047 (a)	0.5071 ± 0.122 (a)	0.2685 ± 0.049 (a)	5.109 ± 1.727 (a)	0.680 ± 0.195 (a)	477.58 ± 49.36 (a)	97.42 ± 40.44 (b)	380.17 ± 31.19 (a)	258.84 ± 25.61 (a)	131.33 ± 31.02 (a)	5.209 ± 3.088 (b)	0.688 ± 0.245 (a)
**S/MAY**	0.8069 ± 0.032 (c)	0.6839 ± 0.048 (a)	0.5535 ± 0.044 (a)	0.5164 ± 0.097 (a)	0.2664 ± 0.052 (a)	5.312 ± 2.104 (a)	0.699 ± 0.178 (a)	480.58 ± 35.97 (a)	95.85 ± 22.11 (b)	384.73 ± 22.55 (a)	268.69 ± 21.88 (a)	136.04 ± 26.85 (a)	5.595 ± 2.166 (b)	0.718 ± 0.193 (a)
**SG/OCT**	0.7523 ± 0.051 (d)	0.5848 ± 0.060 (b)	0.4450 ± 0.056 (c)	0.5324 ± 0.134 (a)	0.2350 ± 0.073 (b)	2.456 ± 1.290 (c)	0.346 ± 0.234 (c)	434.91 ± 90.10 (b)	108.96 ± 37.59 (a)	325.95 ± 61.57 (c)	192.26 ± 36.12 (d)	103.69 ± 31.93 (b)	29.409 ± 27.861 (a)	0.457 ± 0.279 (c)

Note: Data are presented as mean ± standard deviation (sd). Each group had 10 biological replicates. Letters in parentheses indicate significant differences between treatments according to Tukey's test ($\alpha = 0.05$) (a > b > c).
